# Functional evaluation of circulating hematopoietic progenitors in Noonan syndrome

**DOI:** 10.3892/or.2013.2535

**Published:** 2013-06-11

**Authors:** FABIO TIMEUS, NICOLETTA CRESCENZIO, GIUSEPPINA BALDASSARRE, ALESSANDRA DORIA, STEFANO VALLERO, LUISELDA FOGLIA, SARA PAGLIANO, CESARE ROSSI, MARGHERITA CIRILLO SILENGO, UGO RAMENGHI, FRANCA FAGIOLI, LUCA CORDERO DI MONTEZEMOLO, GIOVANNI BATTISTA FERRERO

**Affiliations:** 1 Pediatric Hematology-Oncology, Regina Margherita Children’s Hospital, 10126 Turin, Italy; 2 Department of Pediatrics, University of Turin, 10126 Turin, Italy; 3 Department of Genetics, University of Bologna, Policlinico S. Orsola-Malpighi, 40138 Bologna, Italy

**Keywords:** Noonan syndrome, juvenile myelomonocytic leukemia, CD34^+^ cells, CFU-GM

## Abstract

Noonan syndrome (NS) is an autosomal dominant disorder, characterized by short stature, multiple dysmorphisms and congenital heart defects. A myeloproliferative disorder (NS/MPD), resembling juvenile myelomonocytic leukemia (JMML), is occasionally diagnosed in infants with NS. In the present study, we performed a functional evaluation of the circulating hematopoietic progenitors in a series of NS, NS/MPD and JMML patients. The different functional patterns were compared with the aim to identify a possible NS subgroup worthy of stringent hematological follow-up for an increased risk of MPD development. We studied 27 NS and 5 JMML patients fulfilling EWOG-MDS criteria. The more frequent molecular defects observed in NS were mutations in the PTPN11 and SOS genes. The absolute count of monocytes, circulating CD34^+^ hematopoietic progenitors, their apoptotic rate and the number of circulating CFU-GMs cultured in the presence of decreasing concentrations or in the absence of granulocyte-macrophage colony-stimulating factor (GM-CSF) were evaluated. All JMML patients showed monocytosis >1,000/μl. Ten out of the 27 NS patients showed monocytosis >1,000/μl, which included the 3 NS/MPD patients. In JMML patients, circulating CD34^+^ cells were significantly increased (median, 109.8/μl; range, 44–232) with a low rate of apoptosis (median, 2.1%; range, 0.4–12.1%), and circulating CFU-GMs were hyper-responsive to GM-CSF. NS/MPD patients showed the same flow cytometric pattern as the JMML patients (median, CD34^+^ cells/μl, 205.7; range, 58–1374; median apoptotic rate, 1.4%; range, 0.2–2.4%) and their circulating CFU-GMs were hyper-responsive to GM-CSF. These functional alterations appeared 10 months before the typical clinical manifestations in 1 NS/MPD patient. In NS, the CD34^+^ absolute cell count and circulating CFU-GMs showed a normal pattern (median CD34^+^ cells/μl, 4.9; range, 1.3–17.5), whereas the CD34^+^ cell apoptotic rate was significantly decreased in comparison with the controls (median, 8.6%; range, 0–27.7% vs. median, 17.6%; range, 2.8–49.6%), suggesting an increased CD34^+^ cell survival. The functional evaluation of circulating hematopoietic progenitors showed specific patterns in NS and NS/MPD. These tests are a reliable integrative tool that, together with clinical data and other hematological parameters, could help detect NS patients with a high risk for a myeloproliferative evolution.

## Introduction

Noonan syndrome (NS) is an autosomal dominant disorder, mainly characterized by proportionate short stature, facial and muscoloskeletal dysmorphisms and congenital heart defects (most commonly pulmonary valve stenosis and hypertrophic cardiomiopathy) with an incidence rate between 1:1,000 and 1:2,500 live births (1). Further findings may include cryptorchidism, bleeding diathesis, lymphatic dysplasia and mild to moderate developmental delay/intellectual disability (DD/ID). A myeloproliferative disorder (NS/MPD) can occasionally be diagnosed in infants with NS. The clinical course of NS/MPD is usually benign with spontaneous remission. However, various cases with an aggressive course resembling juvenile myelomonocytic leukemia (JMML) have been described (2–6). JMML is a rare clonal myelodysplastic-myeloproliferative disorder typical of infancy and early childhood, characterized by spontaneous *in vitro* proliferation of bone marrow and peripheral blood hematopoietic progenitors in the absence of exogenous growth factors, due to selective hypersensitivity to granulocyte-macrophage colony-stimulating factor (GM-CSF) (7). Hepatosplenomegaly, lymphoadenopathy, anemia, thrombocytopenia, and fever, variably associated with symptoms of non-hematopoietic organ infiltration, are common clinical findings in JMML. The fulfillment of the following laboratory criteria is required for JMML diagnosis: an absolute monocyte count >1,000/μl, <20% bone marrow blasts and the absence of t (9;22) or BCR/ABL rearrangement. Apart from such mandatory criteria, JMML patients may present with a high white blood cell count (>10,000/μl), immature myeloid precursors on a peripheral smear and increased fetal hemoglobin (HbF) for age. Monosomy 7 is quite frequently noted (8).

Both NS and JMML are characterized by hyperactivation of the RAS/MAPK signaling pathway. In NS, germline missense mutations in genes such as PTPN11, KRAS, SOS1, RAF1, BRAF, SHOC2, NRAS and CBL (9–19) have been documented. In JMML, mutually exclusive somatic mutations of PTPN11, KRAS, NRAS and NF1 genes can be found in ~75% of cases. NS is associated with germline PTPN11 mutations in ~50% of the patients, while somatic PTPN11 mutations are found in 35% of children with JMML. This gene encodes for the ubiquitously expressed non-receptor protein tyrosine phosphatase (PTP) SHP-2, which is implicated in a variety of intracellular signaling cascades mediated by growth factors, cytokines, hormones and cell adhesion molecules (20,21). SHP-2 is also involved in several developmental processes, in particular semi-lunar valvulogenesis (22) and hematopoietic cell differentiation (23,24). PTPN11 mutations favor either the basal activity or the response to inducing events of the catalytically active conformation of SHP-2, resulting in gain of function (11). The PTPN11 mutational spectrum has been shown to be different in JMML, NS/MPD and NS without any hematological abnormalities (5).

We previously demonstrated that flow cytometric evaluation of the absolute count of peripheral blood (PB) CD34^+^ cells and the apoptotic rate is a simple and repeatable technique, useful for early detection of clonal evolution in acquired aplastic anemia. In children with *de novo* or secondary refractory anemia with blast excess (RAEB) we observed a typical pattern of a high PB CD34^+^ count associated with a low apoptotic rate, sometimes evident months before the appearance of the complete clinical image (25).

In the present study, we performed a functional evaluation of the circulating hematopoietic progenitors in a series of NS patients. Clonogenic tests in the absence or in the presence of increasing concentrations of GM-CSF and three-color flow cytometric analysis for CD45, CD34, and Annexin V were performed using the PB of 27 patients with NS and 5 patients with JMML. The different functional patterns were compared to identify a possible NS subgroup worthy of stringent hematological follow-up for an increased risk of MPD development.

## Materials and methods

### Patients

Included in the present study were 27 patients admitted to Department of Pediatrics with a clinical diagnosis of NS (independently of their mutational status and the presence of hematological anomalies). Three patients had a myeloproliferative disorder (NS/MPD), with monocytosis, atypical monocytoid cells, myelodysplastic features and granulocyte precursors in PB, thrombocytopenia and hepato-splenomegaly (2–6). Five patients with a diagnosis of JMML, fulfilling the EWOG-MDS criteria (8) were also studied. PB samples were collected in EDTA at diagnosis. Further blood samples were collected and analyzed in NS patients when hematological anomalies (e.g. anemia, thrombocytopenia, leukocytosis, splenomegaly, lymphadenopathy) and/or alterations of the functional pattern of circulating hematopoietic progenitors were observed. NS/MPD and JMML patients were evaluated at various stages during follow-up and treatment. Ethics committee approval and informed consent of the parents of the patients were obtained.

### Genomic mutational analysis

Genomic DNA was isolated from 200 μl of PB by the QIAamp DNA Blood Mini kit (Qiagen, Germantown, MD, USA). A molecular analysis of PTPN11, KRAS, SOS1, RAF1, BRAF, SHOC2, NRAS and CBL was performed as previously described (11–20).

### Absolute count of CD34^+^ cells and the apoptotic index

Flow cytometric analysis was performed within 2 h after venipuncture. The absolute count of CD34^+^ cells and the apoptotic rate were evaluated by a three-color fluorescence for CD45, CD34 and Annexin V as follows. A total of 5×10^5^ nucleated cells were incubated for 20 min at 4°C with anti-CD34 PE (8G12; Becton-Dickinson, San José, CA, USA) and anti-CD45 PerCP (2D1; BD Biosciences, Franklin Lakes, NJ, USA). After incubation and red cell lysis by ammonium chloride, the samples were washed in cold phosphate-buffered saline (PBS) and incubated with Annexin V-fluorescein isothiocyanate (FITC) (Apoptosis Detection kit; R&D Systems, Minneapolis, MN, USA), according to the manufacturer’s instructions. The cells were then analyzed in a Coulter Epics XL2 (IL, Bedford, MA, USA) cytometer equipped with an argon laser. CD34^+^ cells were identified by a sequential gating strategy according to the ISHAGE protocol (26). Absolute CD34 counts were assessed by a two-platform method with a Sysmex K4500 counter (Sysmex Corporation, Kobe, Japan). At least 100 CD34^+^ cells were evaluated in each experiment.

### Cell cultures

Low-density mononuclear cells (2×10^5^) obtained from the patient PB by density centrifugation over Ficoll-Hypaque gradient were plated in multi well plates in 250 μl Iscove’s modified Dulbecco’s medium (IMDM) containing 30% fetal calf serum (FCS) (both from Sigma-Aldrich, St. Louis, MO, USA), 0.3% noble agar and 100 U/ml rhIL-3 and decreasing concentrations (20, 10, 5, 1, 0.1 ng/ml) of rhGM-CSF (both from Invitrogen Life Technologies, Carlsbad, CA, USA). After 14 days, single aggregates of >40 cells were scored as CFU-GMs. GM-CFU assay was also performed without GM-CSF stimulation. GM-CFU assay in the same culture conditions was also performed in 21 pediatric controls (median age, 9.0; range, 1–18 years).

### Statistical analysis

The patients were divided into 3 groups: NS, NS/MPD and JMML. Historical controls (n=68) from our laboratory were utilized for the absolute count of CD34^+^ in PB and the apoptotic rate (25).

For the absolute count of CD34^+^ cells and apoptotic rate, the values for each patient were compared with the mean control value adjusted for age, as previously published (25).

The cell culture data and the absolute count of CD34^+^ cells and the apoptotic rate results were analyzed using the non-parametric Kruskal-Wallis test. Pairwise comparisons for the disease groups were performed for each of the GM-CSF concentration utilized in the cell cultures, as well as for the 4 variables analyzed in the test (absolute CD34^+^, percentage of CD34^+^/Annexin V^+^, absolute CD34^+^ to the mean age group value ratio, percentage of CD34^+^/Annexin V^+^ to the mean age group value ratio). Fisher’s exact probability test was utilized for correlations between the groups.

## Results

### Mutational spectrum of the patients

The mutational spectrum of our series of NS, NS/MPD and JMML patients is shown in Table I.

### Monocyte counts

All JMML patients showed monocytosis >1,000/μl. Ten out of the 27 NS patients showed monocytosis >1,000/μl, which included the 3 NS/MPD patients (Tables I and II).

### Platelet counts

All JMML patients showed thrombocytopenia as well as 1 out of the 3 NS/MPD patients. In the other NS patients, the platelet counts were in the range of normality, without a correlation with monocyte counts (R=0.016) (Table I).

### Absolute CD34^+^ cell count and apoptotic rate

The PB absolute CD34^+^ cell counts and apoptotic rates in the different groups of patients are shown in Tables I and II. In JMML and NS/MPD patients, we observed high levels of circulating CD34^+^ cells with a low apoptotic rate. In NS patients, CD34^+^ cell counts were normal, whereas their apoptotic rate was significantly lower than that in the controls (p<0.01). Concerning the absolute CD34^+^cell count, statistically significant differences were noted among the NS and JMML (p=0.001), NS and NS/MPD (p<0.05), controls and JMML (p<0.01), and controls and NS/MPD patients (p<0.05). In contrast, no differences in the absolute CD34^+^ cell count were observed between the controls and NS or between the NS/MPD and JMML patients. Normalizing the absolute CD34^+^cell count for age (absolute CD34^+^ to mean age group value ratio), the results from the pairwise comparison did not change.

A pairwise comparison of the Annexin V^+^ percentage showed a statistically significant decrease in the apoptotic rate in each disease group when compared with the controls: NS (p<0.01), NS/MPD (p<0.05), JMML(p<0.01); whereas no significant difference was observed between NS/MPD and JMML, JMML and NS, and NS/MPD and NS patients. When the percentage of Annexin V^+^ cells was normalized for age (percentage of Annexin V^+^/CD34^+^ cells to mean age group value ratio), the results for the pairwise comparison did not change.

### Cell cultures

In the JMML patients, the clonogenic assays from PB showed hypersensitivity to GM-CSF and spontaneous CFU-GM growth (except in one patient previously treated with chemotherapy at another center who did not show spontaneous CFU-GM growth) (Table I). In 3 out of 3 NS/MPD patients we observed hypersensitivity to GM-CSF (in patient NS/MPD3 during the follow-up) and in 2 out of 3 spontaneous CFU-GM growth was noted. In 2 NS patients (NS13 and NS19) we observed hypersensitivity to GM-CSF, and no spontaneous CFU-GM growth. One NS patient (NS11) showed hypersensitivity to GM-CSF and spontaneous CFU-GM growth. This patient had a favorable clinical outcome, and the clonogenic assays performed 6 months later showed normal results.

We observed a significant difference in the distribution and in the median values of CFU-GM among the 3 groups (JMML, NS, NS/MPD) (Table II). Table III (pairwise comparisons of GM-CSF-stimulated clonogenic assays) showed that at different GM-CSF concentrations, JMML patients were significantly more responsive to GM-CSF than both the controls and NS patients. Concerning unstimulated cultures, a significant growth advantage was observed also in NS/MPD when compared with the controls and NS patients (Table III). Specifically, clonogenic assays without GM-CSF were able to distinguish between NS and NS/MPD patients. Fisher’s exact probability test showed a significant correlation between the groups in the unstimulated colony growth tests (0% in controls, 4.2% in NS, 80% in JMML, 66.7% in NS/MPD, p<0.001).

### Follow-up of NS and NS/MPD patients

Six NS patients showed isolated monocytosis >1,000/μl, 2 NS patients showed isolated hyper-responses to GM-CSF, 5 NS patients showed an isolated increase in circulating CD34^+^ cells. In all of these patients a 12-month follow-up showed no alterations in clinical findings. Patients NS11 and NS/MPD1 showed monocytosis >1,000/μl, a hyper-response to GM-CSF, CFU-GM growth without GM-CSF stimulation, high circulating CD34^+^ counts with a low apoptotic rate. In these 2 NS patients a clinical and laboratory follow-up was performed. Patient NS11 carrying the Glu76Asp PTPN11 mutation, was phenotypically characterized by polyhydramnios in the prenatal history, typical facial dysmorphisms, pulmonic stenosis, bilateral cryptorchidism and normal neuropsychomotor development. His clinical follow-up in the following months was normal, and a laboratory hematologic evaluation performed 12 months later showed normal response to GM-CSF, no spontaneous colony growth and normal CD34^+^ cell and monocyte counts.

Patient NS/MPD1 was first evaluated at the age of 2 months while she was in the cardiac surgery unit due to obstructive hypertrophic cardiomyopathy. A clinical diagnosis of NS was confirmed by the molecular analysis of the PTPN11 gene, that revealed the Phe285Ser mutation. A preliminary hematological evaluation evidenced only mild hepatomegaly and monocytosis. Clonogenic assays and flow cytometry showed hypersensitivity to GM-CSF, spontaneous CFU-GM growth, increased circulating CD34^+^ cells with a low apoptotic rate. At the age of 12 months, the patient showed splenomegaly, thrombocytopenia and monocytosis, with the presence in the peripheral blood smear of 10% of atypical monocytoid cells, moderate myelodysplastic features and granulocyte precursors. Bone marrow aspirate showed mild myelodysplasia and the presence of 15% of atypical monocytoid elements. A polymerase chain reaction (PCR) for bcr/abl rearrangement was negative. The HUMARA assay performed on peripheral blood populations was normal, excluding a frank JMML evolution and suggesting a polyclonal myeloproliferative disorder, described in NS (2). Hence, a diagnosis of NS/MPD was made. Thrombocytopenia and splenomegaly persisted over the following months, and the clinical course worsened, with a progressive development of lymphatic dysplasia with thoracic duct ectasia, progressive severe respiratory insufficiency, pleural effusion and exitus at the age of 20 months for acute cardiopulmonary failure.

**Figure 1:**
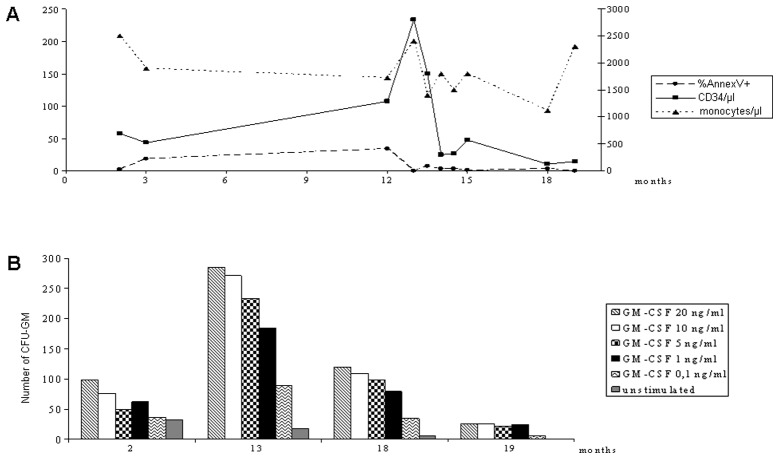
Detailed functional follow-up of patient NS/MPD1. (A) Peripheral blood absolute CD34^+^ cell counts (CD34/μl) and apoptotic rate (% Annexin V^+^) and absolute monocyte counts (monocytes/μl) are shown. (B) Numbers of peripheral blood CFU-GMs cultured with decreasing concentrations of GM-CSF or without GM-CSF stimulation (unstimulated) are shown.

Patients NS/MPD2 and NS/MPD3 were diagnosed in the first month of life presenting a myeloproliferative disorder. Table IV shows the clinical and laboratory follow-up of the NS/MPD patients, and Fig. 1 shows a detailed follow-up of patient NS/MPD1.

## Discussion

Genetic diseases associated with a high tumor risk are models for the study of carcinogenesis. A close correlation is often observed between such genetic conditions and specific acquired neoplastic diseases. NS and JMML were shown to be strictly correlated to each other. Indeed, the same signal transduction pathway (RAS/MAPK) is hyperactivated both in NS and in JMML, with an involvement of the same genes. Moreover, NS patients presented an increased risk of developing JMML or, more frequently, a transient myeloproliferative disorder associated with Noonan syndrome (NS/MPD).

Strictly correlated to the hyperactivation of the RAS/MAPK pathway is the hypersensitivity to GM-CSF observed in JMML (7).

In the present study, we conducted a molecular study of a cohort of NS and JMML patients with a functional evaluation of their circulating hematopoietic progenitors, and correlated the results with the clinical-hematological course. In particular, our aim was to evaluate the circulating CD34^+^ cell count and apoptotic rate and to relate such findings with *in vitro* colony growth (in the absence and with increasing concentrations of GM-CSF), hematological features and the clinical history of each patient. The analyses were performed on PB, even though the biological variability of hematopoietic progenitor counts and clonogenic assays in PB is higher than in bone marrow. A bone marrow aspirate would have been unethical in NS patients without any sign of a hematological disease.

Even though a constitutional GM-CSF hypersensitivity has been suggested in NS (6,27), we observed hypersensitivity to GM-CSF in only 6 of the 27 NS patients. One of them developed a myeloproliferative disorder 12 months later (NS/MPD1) and 2 patients (NS/MPD2 and NS/MPD3) had a transient myeloproliferative syndrome at the time of the study. In patient NS/MPD3, a hyper-response to GM-CSF was observed during the follow-up, but not at diagnosis. The other 3 NS patients with GM-CSF hypersensitivity had normal hematological profiles. These data suggest that the response to GM-CSF is variable in NS patients.

PTPN11 mutations in JMML affect amino acids differently from those involved in NS (5,28,29). Somatic JMML-associated mutations are predicted to result in a stronger SHP-2 gain of function than germ-line mutations described in NS, and the leukemic transformation in NS seems related to cooperating molecular lesions (5). In our NS series, one patient (NS5) carried the Gly503Glu PTPN11 mutation, also described in JMML. Interestingly, this patient showed a normal hematological profile without hypersensitivity to GM-CSF, but his mother, who presented with a short stature and typical facial appearance, as revealed by anamnestic data and family photographs, died due to non-Hodgkin lymphoma.

In a previous study, we observed in normal subjects a progressive decrease in circulating CD34^+^ cells and a progressive increase in their apoptotic rate from the first months of life to adult age (25). An increase in circulating CD34^+^ cells has been described in myelofibrosis with myeloid metaplasia in adults (30) and in RAEB in adults (31) and in children, associated with a low apoptotic rate (25). Here, we observed a similar behavior in the JMML and in the NS/MPD patients. PB CD34^+^ cell counts in the majority of our NS patients were normal, but the apoptotic CD34^+^ cell rate was significantly lower than that in the controls, as in JMML and NS/MPD. Previous studies have pointed to an increased proliferative activity of hematopoietic progenitors in NS. Our results allow us to identify NS as a disease with a lower-than-normal apoptotic activity of circulating hematopoietic progenitors. This increase in the survival of hematopoietic progenitors appears to be a hallmark of NS patients.

Two out of 3 NS/MPD patients shared the same functional pattern of JMML, characterized by high circulating CD34^+^ cell counts with a very low apoptotic rate, hypersensitivity to GM-CSF and spontaneous CFU-GM growth.

The present data represent the complex hematopoietic functional profile of a series of NS patients and suggest that the evaluation of the absolute CD34^+^ count and the apoptotic rate as well as CFU-GM assay performed on PB could be a non-invasive and repeatable method that can facilitate the identification of NS patients with a higher risk of myeloproliferative evolution for whom an intensified hematological follow-up program is justified.

## Figures and Tables

**Table I:** Mutational spectrum, WBC, PLT and monocyte counts, CD34^+^ absolute count and apoptotic rate, CFU-GM from peripheral blood in a series of NS, NS/MPD and JMML patients.

Patient	Mutated gene	Mutation	Hypersensitivity to GM-CSF	Unstimulated colony growth	Annexin V^+^/CD34^+^ (%)		CD34 (/μl)		WBC (10^3^/μl)	Monocytes (10^3^/μl)	PLT (10^3^/μl)
NS1	PTPN11	Gln79Arg	No	No	8.5	L	3.6	N	11.9	0.740	284
NS2	PTPN11	Asn58His	No	No	26.1	H	7	N	11.6	0.400	262
NS3	PTPN11	Tyr63Cys	No	No	16.1	N	2.5	L	8.3	0.300	428
NS4	PTPN11	Gly503Arg	No	No	2.56	L	3.42	L	17.1	1.720	331
NS5	PTPN11	Gly503Glu	No	No	7.6	L	3.6	N	7.3	0.900	249
NS6	PTPN11	Asn308Ser	No	No	12	L	6.2	H	10.3	0.690	297
NS7	PTPN11	Leu261His	No	No	10	L	2.9	N	5.8	0.470	416
NS8	PTPN11	Leu261His	No	No	0	L	4.8	N	12.0	0.770	376
NS9	PTPN11	Leu261His	No	No	10	L	17.52	H	7.3	0.600	277
NS10	PTPN11	Phe285Ile	No	No	7.9	L	3.8	N	7.7	0.410	218
NS11	PTPN11	Glu76Asp	Yes	Yes	1.0	L	12	H	12.0	1.140	300
NS12	PTPN11	Glu139Asp	No	No	17.8	L	2.2	L	7.3	0.510	198
NS13	PTPN11	Asp61Asn	Yes	No	4.1	L	13.8	H	15.3	0.840	212
NS14	SOS	Ile252Thr	No	No	27.7	H	2.6	L	6.4	0.450	259
NS15	SOS	Thr266Lys	No	No	8.8	L	16.2	H	12.5	0.620	291
NS16	SOS	Arg552Gly	No	No	18.2	L	1.3	L	4.3	0.340	259
NS17	SOS	Glu433Lys	No	No	0	L	7.75	N	15.5	1.420	422
NS18	RAF1	Ser257Leu	No	No	8	L	2.5	L	6.4	0.540	253
NS19	SOS	Met269Thr	Yes	No	4.4	L	5.2	N	8.7	1.100	182
NS20	n.d.	n.d.	No	No	11.2	N	4.5	L	12.2	1.110	184
NS21	KRAS	Gln22Arg	No	No	15.7	L	5.6	N	9.44	0.630	551
NS22	BRAF	Leu597Val	No	No	0.09	L	7.1	H	7.9	1.600	397
NS23	RAF1	Pro261Ser	No	No	25	H	5	N	12.6	1.450	384
NS24	SHOC2	Ser2Gly	No	No	6.8	L	8.5	N	17.0	0.700	482
NS/MPD1	PTPN11	Phe285Ser	Yes	Yes	2.4	L	58.0	H	14.5	2.500	399
NS/MPD2	PTPN11	Asp61Asn	Yes	Yes	0.2	L	1,374	H	43.5	5.520	54
NS/MPD3	PTPN11	Asp61Asn	Yes	No	1.6	L	205.7	H	20.2	2.550	167
JMML1	n.d.	n.d.	Yes	Yes	2.1	L	193.6	H	9.3	1.900	55
JMML2	PTPN11	Glu76Gly	Yes	Yes	12.1	N	44	H	8.8	1.380	31
JMML3	PTPN11	Gly503Val	Yes	No	7.2	L	49.6	H	3.1	1.070	45
JMML4	NF1	n.d.	Yes	Yes	0.4	L	109.8	H	11.2	1.430	15
JMML5	n.m.	n.d.	Yes	Yes	0.4	L	232	H	36.2	7.610	71

NS, Noonan syndrome patients (n=24); NS/MPD, Noonan syndrome patients with myeloproliferative evolution (n=3); JMML, juvenile myelomonocytic leukemia patients (n=5). For ‘% AnnexinV^+^/CD34^+^’ and ‘CD34^+^/μl’ categories: ‘L’ (low), indicated samples in which the values of Annexin V expression on CD34^+^ cells or the CD34^+^ absolute count were lower than the average −2 SD, compared to the reference values for the same age groups (reviewed in ref. 25); ‘N’ (normal) indicated samples in which the value was included in the average values ± 1 SD; ‘H’ (high) indicated samples in which the value was higher than the average +2 SD. Patient NS/MPD3 developed GM-CSF hypersensitivity during follow-up (Table IV). WBC, white blood count; PLT, platelets. n.d., not determined; n.m., no mutation.

**Table II:** Circulating monocytes, peripheral blood CD34^+^ cells, their apoptotic rate and CFU-GMs in a series of NS, NS/MPD and JMML patients.

Clinical parameters	Controls	NS	NS/MPD	JMML
Monocytes (/μl)	600 (200–900)	695 (300–1,720)	2,550 (2,500–5,520)	1,600 (1,070–7,600)
CD34^+^ (/μl)	5.2 (1.8–23.1)	4.9 (1.3–17.5)	205.7 (58–1,374)	109.8 (44–232)
Annexin V^+^/CD34^+^ (%)	17.6 (2.8–49.6)	8.6 (0.0–27.7)	1.4 (0.2–2.4)	2.1 (0.4–12.1)
GM-CSF (20 ng/ml)	9 (0–26)	4 (0–34)	36 (0–84)	46 (18–258)
GM-CSF (10 ng/ml)	5 (0–18)	2 (0–30)	38 (0–76)	40 (10–244)
GM-CSF (5 ng/ml)	3 (0–18)	1 (0–24)	38 (0–50)	42 (6–262)
GM-CSF (1 ng/ml)	1 (0–16)	1 (0–23)	34 (0–62)	32 (6–264)
GM-CSF (0.1 ng/ml)	0 (0–14)	0 (0–8)	26 (0–36)	30 (2–240)
Unstimulated	0 (0–0)	0 (0–4)	4 (0–32)	18 (0–84)

NS, Noonan syndrome patients (n=24); NS/MPD, Noonan syndrome patients with myeloproliferative evolution (n=3); JMML, myelomonocytic leukemia patients (n=5). CFU-GMs from 2×10^5^ circulating mononuclear cells were cultured in the presence of decreasing GM-CSF concentrations and without GM-CSF (unstimulated). Data are provided as mean value (range).

**Table III:** Pairwise comparisons of the GM-CSF-stimulated clonogenic assay in the disease groups.

Pair	GM-CSF (20 ng/ml)	GM-CSF (10 ng/ml0	GM-CSF (5 ng/ml)	GM-CSF (1 ng/ml)	GM-CSF (0.1 ng/ml)	Unstimulated
Controls-NS	0.248	1.000	1.000	1.000	1.000	1.000
Controls-NS/MPD	1.000	1.000	1.000	1.000	0.460	**0.010**
Controls-JMML	0.079	**0.038**	**0.022**	**0.010**	**0.007**	**0.000**
NS-JMML	**0.001**	**0.002**	**0.004**	**0.004**	**0.003**	**0.000**
NS-NS/MPD	0.671	0.808	0.903	0.786	0.674	**0.016**
NS/MPD-JMML	1.000	1.000	1.000	1.000	1.000	1.000

NS, Noonan syndrome patients, (n=24); NS/MPD, Noonan syndrome patients with myeloproliferative evolution, (n=3); JMML, myelomonocytic leukemia patients, (n=5). Bold print indicates statistically significant p-values (p<0.05; Kruskal-Wallis test). CFU-GM from circulating mononuclear cells cultured in the presence of decreasing GM-CSF concentrations or without GM-CSF (unstimulated).

**Table IV:** Functional follow-up of 3 NS/MPD patients with myeloproliferative evolution.

	NS/MPD1		NS/MPD2		NS/MPD3	
			
Parameters	T_0_	T_1_	T_0_	T_1_	T_0_	T_1_
Age at T_0_	2 months		10 days		1 month	
WBC (/μl)	14,500	8,200	43,500	8,480	20,200	16,000
Monocytes (/μl)	2,500	1,400	5,520	620	2,550	1,280
CD34^+^ (/μl)	58	150	1,374	13	205	45
Annexin V^+^ (%)	2.4	7.5	0.2	0.8	1.6	3.0
Hyper-response to GM-CSF	+++	++	+++	No	No	+++
Spontaneous CFU growth	++	++	+	No	No	No
Circulating dysplastic monocytes with myeloid dysplasia	No	+++	++	No	+	No
Hepatosplenomegaly	No	++	No	No	++	No
Thrombocytopenia	No	+++	++	No	No	No

NS/MPD, Noonan syndrome patients with myeloproliferative evolution. T_0_, first observation; T_1_, observation at 12 months.
